# Draft Genome Sequence of *Pseudomonas fluorescens* ML11A, an Endogenous Strain from Brook Charr with Antagonistic Properties against *Aeromonas salmonicida* subsp. *salmonicida*

**DOI:** 10.1128/genomeA.01716-16

**Published:** 2017-03-02

**Authors:** Jeff Gauthier, Steve J. Charette, Nicolas Derome

**Affiliations:** a Département de Biologie, Institut de Biologie Intégrative et des Systèmes (IBIS), Université Laval, Québec, Canada; b Centre de Recherche de l’Institut Universitaire de Cardiologie et de Pneumologie de Québec (CRIUCPQ), Québec, Canada

## Abstract

*Pseudomonas fluorescens* ML11A, isolated from brook charr, showed a strong *in vitro* inhibitory effect against *Aeromonas salmonicida* subsp. *salmonicida*, a bacterial fish pathogen. Its genome harbors gene clusters for siderophore and bacteriocin biosynthesis and shares 99% whole-genome identity with *P. fluorescens* A506, a biological control strain used in agriculture.

## GENOME ANNOUNCEMENT

*Aeromonas salmonicida* subsp. *salmonicida* is an opportunistic pathogen of farmed salmonid fish with acute episodes resulting in fatal septicemia (1). An increasing number of strains of this bacterium are resistant to multiple antibiotics (2–5). Alternative control and prevention strategies are needed to limit the propagation of antimicrobial resistance (6). Since the host microbiota plays a major role in mitigating colonization and invasion by pathogens (7, 8), administering beneficial bacteria from the microbiota to susceptible hosts appears to be a promising solution against furunculosis, as proven in other host–pathogen combinations (9–12).

A bacterial strain, ML11A, was recovered from brook charr (*Salvelinus fontinalis*) skin mucus (Pisciculture de la Jacques-Cartier Inc., Cap-Santé, QC, Canada). This strain was initially identified as *Pseudomonas* sp. based on 16S rRNA homology with *P. azotoformans* (13). *Pseudomonas* sp. ML11A showed a strong *in vitro* diffusible inhibitory effect against 10 *A. salmonicida* subsp. *salmonicida* strains from North America and Europe. To further investigate the biological safety of *Pseudomonas* sp. ML11A for future use in fish farms and its mechanism of action against *A. salmonicida* subsp. *salmonicida*, its genome was sequenced.

Whole-genome shotgun paired-end libraries (2 × 300 bp) were prepared with a KAPA Hyper Prep Kit (Kapa Biosystems, Wilmington, MA, USA) and sequenced on an Illumina MiSeq sequencer (Illumina, San Diego, CA, USA). Sequence reads were quality-filtered, trimmed, and *de novo* assembled with the A5 pipeline (14). A total of 75 contigs were generated with an average coverage of 22.6-fold. The average contig size was 88,741 bp, and the *N*_50_ contig size was 176,212 bp. The size of the assembled genome is 6,655,593 bp with a G+C content of 59.5%, within range of known values for *Pseudomonas* genomes (15).

*Pseudomonas* sp. ML11A was subsequently identified as *P. fluorescens* on the basis of 99.2% shared average nucleotide identity (16) with *P. fluorescens* A506 (17), a strain approved in the United States and Canada as a biocontrol agent against fire blight of apple and pear (*Erwinia amylovora*) (18). The draft genome of *P. fluorescens* ML11A was annotated using the RAST annotation server (http://rast.nmpdr.org) and the NCBI Prokaryotic Genome Annotation Pipeline (https://www.ncbi.nlm.nih.gov/genome/annotation_prok). It contains 6,054 protein-coding genes, 15 rRNA genes, and 64 tRNA genes. Of all the protein-coding genes, 51% were assigned to 547 SEED subsystems, among which putatively active gene clusters for antibacterial compounds such as colicin V (19) and siderophore pyoverdine (20) were found.

### Accession number(s).

This whole-genome shotgun project has been deposited at DDBJ/ENA/GenBank under the accession number MRXZ00000000. The version described in this paper is the first version, MRXZ01000000.
